# Supplementary material for: Examining Health Literacy in Taiwanese Smoking Cessation Populations: A Multidimensional Evaluation of Sociodemographic Factors and Domain-Specific Competencies
Source: Healthcare (Basel). 2023 Aug 21;11(16):2350. doi: 10.3390/healthcare11162350 (PMC10454805; doi:10.3390/healthcare11162350)
Supplement: Supplementary file 1 [file healthcare-11-02350-s001.zip › healthcare-2457851-supplementary.pdf]

Figure S1 Annex: The HLS-EU-Q47 of the HLS-EU Consortium for the European Health Literacy Survey (HLS-EU)

HLS-EU-Q47

© HLS-EU Consortium

Item no.	Relation to HLS-EU matrix	*On a scale from very easy to very difficult, how easy would you say it is to: …*	1 Very difficult	2 Difficult	3 Easy	4 Very easy	5 (Don't know - to be used by interviewer only)
1	Healthcare/ Access information	find information about symptoms of illnesses that concern you?					
2	Healthcare/ Access information	find information on treatments of illnesses that concern you?					
3	Healthcare/ Access information	find out what to do in case of a medical emergency?					
4	Healthcare/ Access information	find out where to get professional help when you are ill?					
5	Healthcare/ Understand information	understand what your doctor says to you?					
6	Healthcare/ Understand information	understand the leaflets that come with your medicine?					
7	Healthcare/ Understand information	understand what to do in a medical emergency?					
8	Healthcare/ Understand information	understand your doctor's or pharmacist's instruction on how to take a prescribed medicine?					
9	Healthcare/ Appraise information	judge how information from your doctor applies to you?					
10	Healthcare/ Appraise information	judge the advantages and disadvantages of different treatment options?					
11	Healthcare/ Appraise information	judge when you may need to get a second opinion from another doctor?					
12	Healthcare/ Appraise information	judge if the information about illness in the media is reliable?					
13	Healthcare/ Apply information	use information the doctor gives you to make decisions about your illness?					
14	Healthcare/ Apply information	follow the instructions on medication?					
15	Healthcare/ Apply information	call an ambulance in an emergency?					
16	Healthcare/ Apply information	follow instructions from your doctor or pharmacist?					
17	Disease prevention/ Access information	find information about how to manage unhealthy behaviour such as smoking, low physical activity and drinking too much?					
18	Disease prevention/ Access information	find information on how to manage mental health problems like stress or depression?					
19	Disease prevention/ Access information	find information about vaccinations and health screenings that you should have?					
20	Disease prevention/ Access information	find information on how to prevent or manage conditions like being overweight, high blood pressure or high cholesterol?					
21	Disease prevention/ Understand information	understand health warnings about behaviour such as smoking, low physical activity and drinking too much?					
22	Disease prevention/ Understand information	understand why you need vaccinations?					
23	Disease prevention/ Understand information	understand why you need health screenings?					
24	Disease prevention/ Appraise information	judge how reliable health warnings are, such as smoking, low physical activity and drinking too much?					

25	Disease prevention/ Appraise information	judge when you need to go to a doctor for a check-up?	1 Very difficult	2 Difficult	3 Easy	4 Very easy	5 (Don't know - to be used by interviewer only)
Item no.	Relation to HLS-EU matrix	*On a scale from very easy to very difficult, how easy would you say it is to: …*					
26	Disease prevention/ Appraise information	judge which vaccinations you may need?					
27	Disease prevention/ Appraise information	judge which health screenings you should have?					
28	Disease prevention/ Appraise information	judge if the information on health risks in the media is reliable?					
29	Disease prevention/ Apply information	decide if you should have a flu vaccination?					
30	Disease prevention/ Apply information	decide how you can protect yourself from illness based on advice from family and friends?					
31	Disease prevention/ Apply information	decide how you can protect yourself from illness based on information in the media?					
32	Health promotion/ Access information	find information on healthy activities such as exercise, healthy food and nutrition?					
33	Health promotion/ Access information	find out about activities that are good for your mental well-being?					
34	Health promotion/ Access information	find information on how your neighbourhood could be more health-friendly?					
35	Health promotion/ Access information	find out about political changes that may affect health?					
36	Health promotion/ Access information	find out about efforts to promote your health at work?					
37	Health promotion/ Understand information	understand advice on health from family members or friends?					
38	Health promotion/ Understand information	understand information on food packaging?					
39	Health promotion/ Understand information	understand information in the media on how to get healthier?					
40	Health promotion/ Understand information	understand information on how to keep your mind healthy?					
41	Health promotion/ Appraise information	judge where your life affects your health and well-being?					
42	Health promotion/ Appraise information	judge how your housing conditions help you to stay healthy?					
43	Health promotion/ Appraise information	judge which everyday behaviour is related to your health?					
44	Health promotion/ Apply information	make decisions to improve your health?					
45	Health promotion/ Apply information	join a sports club or exercise class if you want to?					
46	Health promotion/ Apply information	influence your living conditions that affect your health and wellbeing?					
47	Health promotion/ Apply information	take part in activities that improve health and well-being in your community?					
